# Genome Assembly of the Ty1-Less Saccharomyces paradoxus Strain DG1768

**DOI:** 10.1128/mra.00868-21

**Published:** 2022-01-20

**Authors:** Jingxuan Chen, Holly McQueary, David W. Hall, Peter Philippsen, David J. Garfinkel, Casey M. Bergman

**Affiliations:** a Institute of Bioinformatics, University of Georgia, Athens, Georgia, USA; b Department of Genetics, University of Georgia, Athens, Georgia, USA; c Molecular Microbiology, Biozentrum, University of Basel, Basel, Switzerland; d Department of Biochemistry and Molecular Biology, University of Georgia, Athens, Georgia, USA; University of Maryland School of Medicine

## Abstract

Here, we report an essentially complete genome assembly for the Ty1-less Saccharomyces paradoxus strain DG1768 (derivative of strain 337) based on PacBio and Illumina shotgun sequence data. We also document the genetic alterations that make this yeast strain a key resource for Ty1 mobility studies.

## ANNOUNCEMENT

*Saccharomyces* yeasts have played essential roles in elucidating the biology of eukaryotic retrotransposons (1–4). Here, we report a genome assembly for Saccharomyces paradoxus strain DG1768, a representative of a “Ty1-less” lineage frequently used to study Ty1 retrotransposition (5–11). DG1768 [*MAT*α *ura3-100 Ty1-155-*Δ*ura3(Scer)-101 his3-*Δ*200hisG*] is derived from a wild *Saccharomyces* strain collected in California (UCDFST 51-186) that was obtained from the Ron Davis laboratory (renamed 155). Michael Ciriacy mated strain 155 with Saccharomyces cerevisiae GRF18 and then repeatedly backcrossed to strain 155 to create a nearly isogenic strain (called 276) carrying *ho(Scer)*, one full-length Ty1 element (called Ty1-155), and a *ura3* mutation at the endogenous *URA3* locus. Homologous recombination was used to replace Ty1-155 in strain 276 with S. cerevisiae *URA3* plus ∼600 bp of vector sequence, followed by selection of a *ura3* mutation in the new heterologous *URA3* locus to create the ρ− strain 337. Finally, *HIS3* was deleted (replaced with Salmonella *HisG*), and functional S. cerevisiae mitochondria were introduced via cytoduction to create strain DG1768 (6). Subsequently, DG1768 was shown to be S. paradoxus (8).

To prepare DNA for sequencing, single colonies of strain DG1768 were inoculated in 7 mL of yeast extract-peptone-dextrose (YPD) liquid broth and cultured for ∼24 h at 30°C. Table 1 provides details on how PacBio and Illumina data sets were generated. For assembly, unfiltered PacBio reads were input to HGAP 4 (smrtlink-release_5.1.0.26412) (12), which performed read quality control, error correction, and adapter trimming. The PacBio-only assembly was polished five times with Pilon (v1.24) (13) using unfiltered paired-end 150-bp Illumina reads as input. Polished contigs were scaffolded with RagTag (v2.0.1) (14) using S. paradoxus YPS138 as the reference (15). Assembly statistics were calculated using QUAST (v5.0.2) (16) and BUSCO (v5.0.0, saccharomycetes_odb10 database) (17). Ty elements were annotated using a RepeatMasker-based pipeline (11). Telomere-associated sequences were annotated with LRSDAY (18). Default software parameters were used except where otherwise noted.

**TABLE 1:** Methods for and properties of the PacBio and Illumina whole-genome sequencing data sets used to assemble and polish the DG1768 genome

Parameter	PacBio reads	Illumina reads
DNA isolation method	Wizard genomic DNA purification kit (Promega)	YeaStar genomic DNA kit (Zymo Research)
Library prepn method	SMRTbell Express template preparation kit >15-kb size-selection protocol with Covaris g-TUBE shearing	Method of Urich et al. (19) with no bisulfite conversion
Sequencing chemistry	Sequel sequencing kit v2.1	S4 reagent kit (300 cycles)
Sequencing instrument	PacBio Sequel II	NovaSeq 6000
Avg read length (bp)	5,912	150
*N*_50_ (bp)	10,652	150
No. of reads	615,827	2,357,654

Our DG1768 assembly is 11,932,051 bp in length (scaffold *N*_50_ value of 917,222 bp), with an overall GC content of 38.43%. The assembly has 18 contigs in 17 scaffolds; 15 chromosomes and the mitochondrial DNA (mtDNA) are completely assembled in 1 contig each, and chromosome XII is assembled in 1 scaffold with 2 contigs, with a gap at the ribosomal DNA (rDNA) locus. A total of 97.5% of Saccharomycetes benchmarking universal single-copy orthologs (BUSCOs) are present and single copy. Core X elements or Y′ elements were annotated in 29 of 32 scaffold ends, indicating the near completeness of all chromosomes. We confirmed that no full-length copies of Ty1 are present in the DG1768 genome. The scar of the Ty1-155 knockout was localized to chromosome VI from nucleotide position 292,634 to 294,347. The scar of the *HIS3* knockout was localized to chromosome XV from nucleotide position 686961 to 688113. We also identified a segment of S. cerevisiae DNA, spanning chromosome IV from nucleotide position 1 to 135828, caused by introduction of the *ho* allele, and we confirmed that DG1768 mtDNA is from S. cerevisiae. The provenance and essentially complete genome assembly for DG1768 presented here should provide a useful resource for studies on Ty1 retrotransposon biology.

### Data availability.

PacBio data used to generate the assembly and Illumina data used to polish the assembly are available under BioProject accession number PRJNA748953. The assembly was deposited at NCBI under GenBank accession numbers CP081969 to CP081986.
